# Graphitic Carbon Nitride Catalyzes the Reduction of the Azo Bond by Hydrazine under Visible Light

**DOI:** 10.3390/nano14171402

**Published:** 2024-08-28

**Authors:** Makobi C. Okolie, Glory G. Ollordaa, Gopal R. Ramidi, Xin Yan, Yufeng Quan, Qingsheng Wang, Yingchun Li

**Affiliations:** 1 Department of Chemistry, Prairie View A&M University, Prairie View, TX 77446, USA; mokolie@student.pvamu.edu (M.C.O.); gollordaa@pvamu.edu (G.G.O.); 2 Department of Chemistry, Texas A&M University, College Station, TX 77843, USA; ramidigopal@tamu.edu (G.R.R.); xyan@tamu.edu (X.Y.); 3 Department of Chemical Engineering, Texas A&M University, College Station, TX 77843, USA; yquan@tamu.edu (Y.Q.); qwang@tamu.edu (Q.W.)

**Keywords:** g-C_3_N_4_, azo, reduction, catalysis, photocatalysis, visible, hydrazine

## Abstract

Graphitic carbon nitride is a semiconducting material of a graphite-like 2D layered structure. It is well known for its photocatalytic properties, which can be exploited for solar-light-driven water splitting and degradation of organic pollutants. Here, we report its capabilities of catalyzing the reduction of the azo bond by hydrazine to two amines under visible light. This photocatalytic reaction provides a novel, appealing way to reduce azo dye wastes as pollutants other than degradation. With this method, the azo dye wastes can be photochemically converted to amines, which can be used as precursors for new azo dyes.

## 1. Introduction

**Figure 1:**
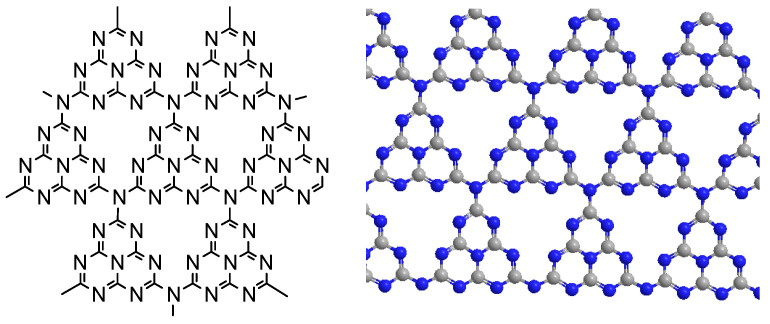
The structure and composition of g-C_3_N_4._

Graphitic carbon nitride (g-C_3_N_4_) has attracted much attention from material scientists due to its interesting 2D graphite-like layered structure, as shown in Figure 1 [1,2]. Such a structure has been expected and proven to have unique electronic properties and applications in various fields, such as hard materials [3], semiconductors [4,5], catalysts [6,7,8], and even as disinfectants [9,10,11]. It has been well documented that g-C_3_N_4_ could be made from different precursors into different morphologies using different methods [12,13,14].

Since the discovery of its photocatalytic capability of splitting water under visible light, most research in the field has been focused on the photocatalytic activity enhancement by design and preparation of novel materials based on it [15,16,17,18]. It has been widely reported that g-C_3_N_4_-based material catalyzes the degradation of organic pollutants in water under visible light [8,19,20]. One such pollutant includes azo dyes, which have been released into the environment as residues from the fabric-dying industry in developing countries [21,22,23]. The degradation of these compounds, which is indicated by color disappearance, has been most often attributed to oxidation by oxygen gas in the air catalyzed by g-C_3_N_4_ under visible light. It is known that the azo bond can be fully reduced to form two amino groups with hydrazine as the reductant in alcoholic solution [24]. This leads to the following questions: (1) Can azo dyes as pollutants in aqueous environments be reduced by hydrazine to amines? (2) If so, can g-C_3_N_4_ act as a photocatalyst to facilitate this reduction process? Positive answers to the questions will provide a method to recycle the azo dye waste. In this recycling process, the azo dye waste will be reduced to amines and the amines can be used to produce azo dyes again via diazonium formation and coupling reactions with aromatic compounds [25,26]. Here we report our study results, which clearly showed that g-C_3_N_4_ catalyzes the reduction of azo dyes to amines by hydrazine under visible light using methyl orange (MO), methyl red (MR), and alizarin yellow (AY) dyes as examples, with structures as shown below:
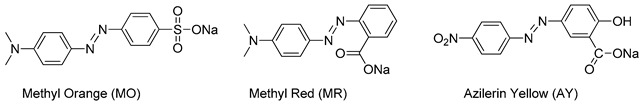


## 2. Experiments

### 2.1. Materials

Photocatalysts (g-C_3_N_4_) were prepared in our lab following a reported method.

Azo dyes: methyl orange (sodium p-dimethylaminoazobenzenesulfonate, MO) was purchased from J.T. Baker Chemical Co., Phillisburg, NJ, USA; methyl red (4-Dimethylaminoazobenzene-2′-carboxylic acid sodium salt, MR) was purchased from Mallinckrodt Baker, Phillisburg, NJ, USA; alizarin yellow R (sodium 2-hydroxy-5-[(E)-(4-nitrophenyl) diazoenyl] benzoate, AY) was purchased from National Aniline Division, Allied Chemical corporation; melamine and hydrazine were purchased from Sigma-Aldrich, St. Louis, MI, USA.

### 2.2. Methods

#### 2.2.1. Methods for the Preparation of Photocatalysts

The photocatalysts used in our experiments were prepared using a previously reported method with slight modification [27]. In general, around 5 g of melamine was ground and transferred into a porcelain crucible. The crucible was then covered with a porcelain cap, put into a muffle furnace (Furnace-Barnstead Thermolyne 1300, Thermo Fisher Scientific Inc., Altoona, IA, USA), heated to the preset temperature, and maintained at that temperature for a total time of 1 to 2 h from the start of heating. After heating was stopped, the furnace cooled down to room temperature and the product in the crucible was collected. The product was ground, characterized, and used as the photocatalysts without further treatment.

#### 2.2.2. Colorimetry Method for Comparison of Photoactivity

Each photocatalyst powder weighed around 10 mg and was placed in its corresponding well on a Pyrex 24-well plate. To each well containing the catalyst, 1 mL of a 2.5% (*v*/*v*) diluted hydrazine aqueous solution and 1 mL azo dye aqueous solution of a concentration of 0.1 mg/mL were added. The reaction mixture was shaken with a Mistral multi-mixer (Mistral Laboratory Equipment Ltd., Los Angeles, CA, USA). After visible light was applied to the solution, pictures of the reaction mixture were taken approximately every 10 min until the color of all solutions disappeared. The faster the color disappears, the more photocatalytic active the catalyst. With this method, the relative activities of our catalysts have been determined.

#### 2.2.3. Kinetic Study of the Photocatalytic and Dark Reactions

The assessment of the photocatalytic activity of g-C_3_N_4_ on the reduction of azo dyes by hydrazine in aqueous solution was carried out with the following method: Into a glass Pyrex petri dish (100 mm in diameter and 20 mm in height) 50.0 mL of deionized water, 0.50 mL of hydrazine (99% in purity), and 25 mg of photocatalyst were added sequentially. The mixture was stirred magnetically at room temperature for 10 min in the dark. Then, 1.50 mL of the stock solution of MO (1.00 mg/mL) was added into the mixture, which was stirred for 5 min. The visible light source (from a 15 w LED light) was turned on and focused on the solution in the petri dish and then timing was started. For every 5 min or 10 min interval since light was shed on the sample, 1 mL of the reaction mixture was taken out and put into a 1.5 mL plastic snap-lock microcentrifuge tube. The suspension was centrifuged at 13,000 rpm for 6–8 min (Eppendorf 5452 Minispin Centrifuge, Eppendorf North America Inc., New York, NY, USA). The clear upper solution in the tube was then transferred into a 1 mL cuvette and its absorbance at 464 nm was measured with a UV spectrometer (Thermo Scientific Spectronic Genesys 20 Spectrophotometer, Thermo Fisher Scientific Inc., Altoona, IA, USA). The plot of the normalized absorbance of the reaction mixture versus the reaction time and curve fitting allowed us to estimate the reaction rate. The dark reaction with and without catalyst, and reaction under light only have also been carried out.

The same method was applied to study the reductions of MR and AY at the same reaction conditions, except that the absorbance was measured at 464 nm and 368 nm, respectively.

The reaction rate of the reduction of each azo dye had been studied with the procedure described above under four different conditions: (1) presence of hydrazine but catalyst and light; (2) presence of hydrazine and catalyst but visible light; (3) presence of hydrazine and visible light but catalyst; and (4) presence of hydrazine, catalyst, and visible light.

#### 2.2.4. Mass Spectrometer and Spectroscopic Method for Detection of Intermediates

Mass spectrometric analysis was performed on an LTQ XL™ linear ion trap mass spectrometer from Thermo Fisher Scientific (San Jose, CA, USA). NanoESI emitters were made from borosilicate glass tubing from WPI Inc. (Sarasota, FL, USA) with a P-100 micropipette puller from Sutter Instrument Company (Novato, CA, USA) using the following parameters: heat, 552; pull, 0; velocity, 8; time, 250; and pressure, 500. Samples were ionized in both positive and negative ion scan modes with spray voltages ranging from 1.5 to 2.5 kV. The following MS parameters were set for data acquisition: the capillary temperature was set at 275 °C; capillary voltage was −50 V and the tube lens was −160 V in the negative ion mode; and capillary voltage was 44 V and the tube lens was 85 V in positive ion mode. Full MS scans were acquired over the range of *m*/*z* 50–800 in both ion modes, the microscan number was set at 2, and the maximum injection time was set at 200 ms.

#### 2.2.5. Method for Powder X-ray Diffraction

Powder X-ray diffraction (PXRD) was performed using a Miniflex II (Rigaku Corporation, The Woodlands, TX, USA) using Cu-Kα radiation (λ = 1.5406 Å) ranging from 5 degrees to 60 degrees.

#### 2.2.6. Method for Scanning Electron Microscopy (SEM)

Scanning electron microscopy (SEM) imaging was conducted on a JSM-7500F (JEOL USA, Inc., Peabody, MA, USA) with a 5 nm coating thickness and 5 keV acceleration voltage.

## 3. Results and Discussion

### 3.1. Synthesis of g-C_3_N_4_ Catalyst

There are several different precursors that lead to g-C_3_N_4_ in a pyrolytic process. We chose melamine as the starting material due to it being easy to access and cheap. By simple pyrolysis, the photocatalysts were prepared at different temperatures from 500 °C to 700 °C and reaction times from 1 to 4 h (Table 1). It was reported that g-C_3_N_4_ is thermally stable at temperatures lower than 500 °C and significantly decomposes at temperature higher than that threshold [27]. The decreasing yields of g-C_3_N_4_ from pyrolysis at increasing temperatures in Table 1 indicates that our pyrolytic products followed the same thermostability trend. Due to significant decomposition of the product at high temperatures, the pyrolysis time was shortened as preparation temperature increased. The color of the products changed from colorless to pale yellow as the temperature increased. The catalyst prepared and its corresponding yield are listed in Table 1. The products were ground to fine powders and used directly for activity assessment without further treatment.

### 3.2. Relative Activity of the Catalysts

**Figure 2:**
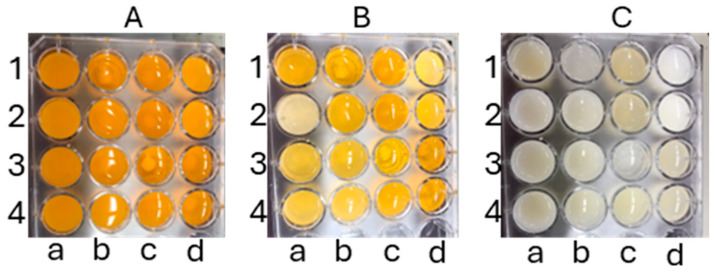
Appearance and color of reaction mixtures in the wells of a plastic plate during exposure to visible light: (**A**) at the beginning of the exposure, (**B**) 5 min after the light exposure, and (**C**) at the end of light exposure.

To measure the relative photocatalytic activity of the catalysts, we carried out a colorimetry assay of catalyst samples with a plate of 24 wells. The process was observed by the naked eyes and photographed at 10 min intervals. The method is illustrated in Figure 2, in which only 16 out of the 24 wells are used. The rows on the plate are labeled by number and columns by letter. The results of one set of our assay are abbreviated into three pictures for comparing and determining the relative photocatalytic activity of the catalysts in the decoloration of MO under visible light. Picture A shows the appearance of the reaction mixtures right before light irradiation. Picture B shows the appearance of the reaction mixtures after about 5 min of light irradiation, which shows that well 2b contained the most active reaction mixture among all those tested and the catalyst was that made at 675 °C. As the reaction proceeded, well 1d then showed the least color among the rest, except for well 2d. The catalyst in well 1d was that made at 650. Following the same principle, the relative activity of the other tested photocatalysts was determined. Picture C shows the reaction mixture when MO in all the wells was reduced to amines after light irradiation for 23 h. The photocatalytic activity of the catalysts was determined to follow this decreasing order: g-C_3_N_4_-700 ≈ g-C_3_N_4_-675 ≈ g-C_3_N_4_-650 > g-C_3_N_4_-625 > g-C_3_N_4_-600 > g-C_3_N_4_-575 > g-C_3_N_4_-550 » C_3_N_4_-525 ≈ g-C_3_N_4_-500 (the number after g-C_3_N_4_ is the temperature in Celsius at which the catalyst was prepared).

The results showed that the activity of the catalysts exhibited strong dependence to the temperature at which it was prepared. In general, the catalysts made at higher temperatures were more active based on the observation that the orange color of the dye faded away much faster with catalysts made at high temperatures than those made at relatively low temperatures. The catalysts made at temperatures of 550 °C or lower were almost inactive. The catalysts made at temperatures of 650 °C or higher had much higher but very similar activity. Due to the significant decomposition above 650 °C, the optimal temperature to prepare the catalyst should be in the range of 625 to 650 °C. Such a colorimetry assay was also applied to MO and AY, and the results obtained were not significantly different from that of MO.

**Figure 3:**
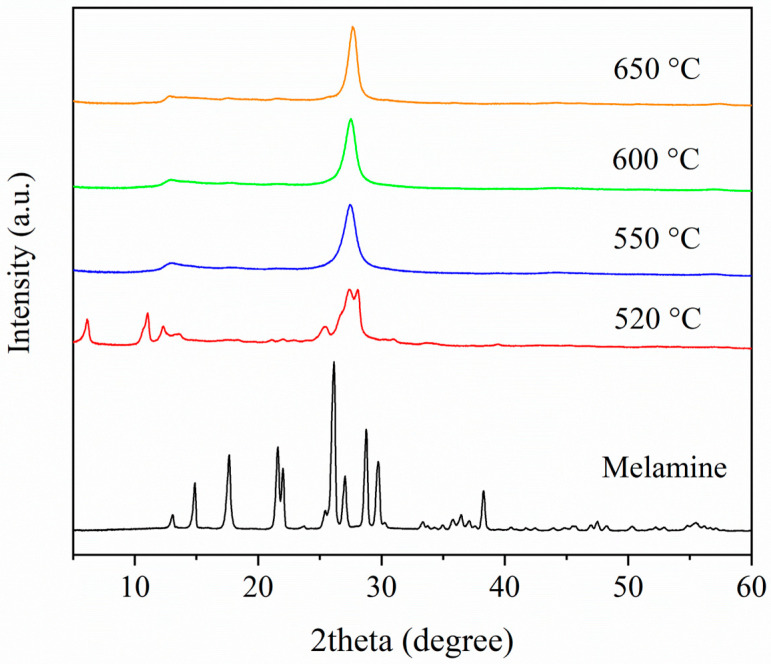
X-ray diffraction patterns of prepared g-C_3_N_4_ catalysts at different temperatures.

To understand the dependence of activity of the catalyst on the temperature, X-ray powder diffraction was applied to the catalysts prepared at 520, 550, 600, and 650 °C, and the results are presented in Figure 3. All three samples made at 550 and above showed two peaks: one is a weak, wide peak around 13.4° and the other is strong at 27.3°, which are attributed to the in-planar structural packing of planes (100) and (002), respectively [28].

**Figure 4:**
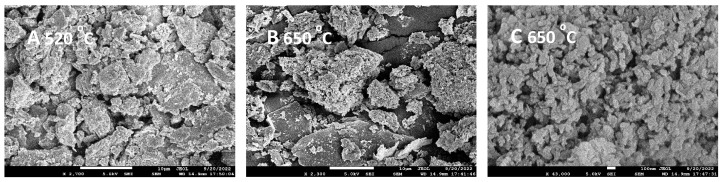
SEM images of catalysts: (**A**) made at 500 °C ×2700; (**B**) made at 650 °C ×2300, and (**C**) made at 650 °C ×43,000.

The two peaks indicated that the catalysts have a typical bulk g-C_3_N_4_ diffraction pattern [29], which is consistent with the graphite-like interlayered structure of heptazine units. Although the three catalysts showed the same XRD pattern, their activity is quite different. The higher activity of catalysts prepared at 650 °C may be attributed to increased defects caused by decomposition at high temperatures. The defects in the crystalline structure have been shown to enhance the photocatalytic property [30]. The catalyst made at 520 °C exhibited very low activity, and it is clearly shown here that its structure is significantly different from those of typical active bulk g-C_3_N_4_. The extra peak may have originated from a melon-a linear polymer of dimelem, which forms from melamine under 500 °C and crosslinks to form the two-dimensional g-C_3_N_4_ at temperature of 550 °C and above [15,31,32]. As the morphology of catalysts can greatly affect the activity, SEM images of the catalysts were collected and are shown in Figure 4.

The grains of bulk g-C_3_N_4_-520 have a layered structure with a smooth surface, while the g-C_3_N_4_-650 grains hardly show any layers in their structure with quite a porous surface. They have irregular short rods interconnected or extruded on the surface. The catalysts made at high temperatures exhibited more uniform pores. Such a porous structure provides a large surface area for interaction between the catalysts and the substrates. This structural feature contributes to the high activity of catalysts made at high temperatures.

**Figure 5:**
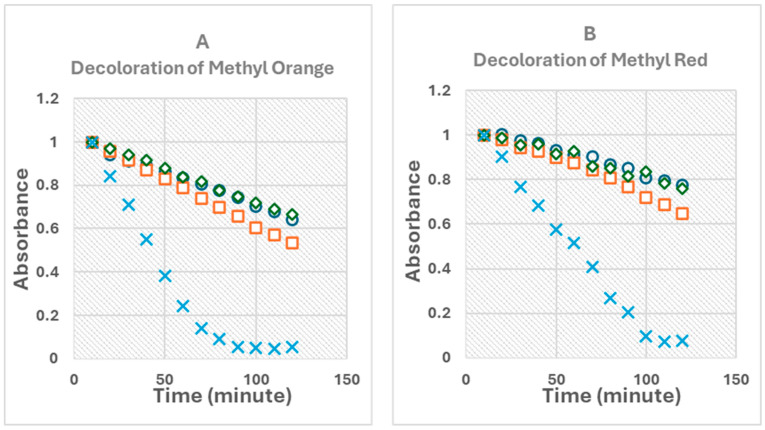
Decoloration rates of azo compounds (**A**) MO and (**B**) MR in hydrazine aqueous solution under different reaction conditions: ○ with no light and no catalyst; **□** with light but no catalyst; ◊ with catalyst but no light; and x with light and catalyst.

To gain a quantitative insight on the photocatalytic effect of g-C_3_N_4_ on the reduction of azo compounds by hydrazine, the decoloration process of MO, MR, and AY were monitored by measurement of absorbance at different times. With the presence of hydrazine, MO and MR exhibited similar decoloration profiles, as shown below in Figure 5.

**Scheme 1:**
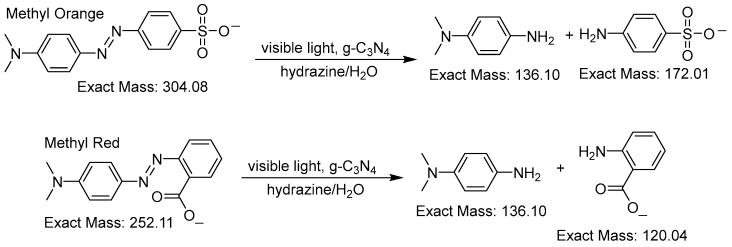
Photocatalytic and dark (when light not applied) reduction of MO and MR by hydrazine and products.

The kinetic profiles indicated the linear relationship between absorbance and time. By observation, it can be concluded that the reactions in the dark with and without the catalyst have the same slope. Therefore, the addition of the catalyst in the dark does not change the decoloration rates of both MO and MR. This means that the catalyst was neither active nor reactive in the dark. The decoloration in the dark is attributed to the reaction of azo compounds with hydrazine. This reduction reaction converted the N=N bond to two amino groups in the products, as shown in Scheme 1.

**Figure 6:**
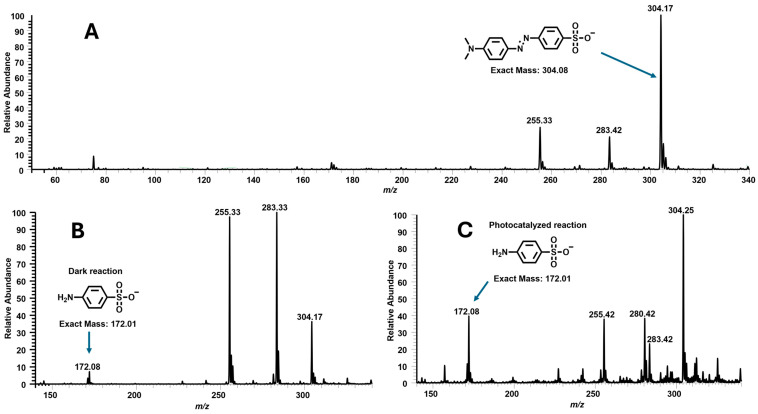
(**A**) MS spectrum of MO 20 min after addition of 3000 equivalent of hydrazine. (**B**) A part of MS spectrum of reaction mixture of MO and 6000 equivalent of hydrazine after 60 min reaction in the dark. (**C**) A part of MS spectrum of reaction mixture of MO with 3000 equivalent of hydrazine 20 min after the photocatalytic reaction under visible light.

In the dark, the reaction proceeded slowly. In contrast, when light was applied, the reactions proceeded a little faster, even without a catalyst. Such results could probably be attributed to light bleaching. The photocatalyzed reaction proceeded with a rate about 5 times faster than the dark reaction for both MO and MR. The initial maximum reaction rates were calculated based on the reaction conditions. The dark reaction rates for MO and MR were measured to be 0.28 μM/min and 0.22 μM/min correspondingly and the photocatalytic reaction rates of 1.3 μM/min and 1.0 μM/min, respectively. The similarity in reaction rates could have originated from the similarity in the structural and electronic structure of MO and MR. To confirm the reaction mechanism and identify the products, the reaction mixtures from different stages were applied to mass spectroscopic analysis. As is shown in Figure 6, the MO anion peak at 304.17 (304.08 calculated) in MS spectrum A was measured to be the dominant anion in the initial photocatalytic reaction mixture. As the reaction proceeded, the intensity of the MR anion peak decreased, as shown in MS spectrum C, and a new peak at 172.08 (172.01 calculated), assigned to p-aminobenzenesulfonate, appeared with increased intensity as the reaction proceeded.

The same mass spectroscopic method was also applied to the dark reduction reaction, and p-aminobenzenesulfonate was also found to be the dominant product in the reaction, as shown in mass spectrum B. When the reaction was completed, as indicated by the formation of a colorless suspension, the MS spectra showed that p-aminobenzenesulfonate was the dominant anion in the reaction mixtures of both the dark and photocatalytic reactions.

**Figure 7:**
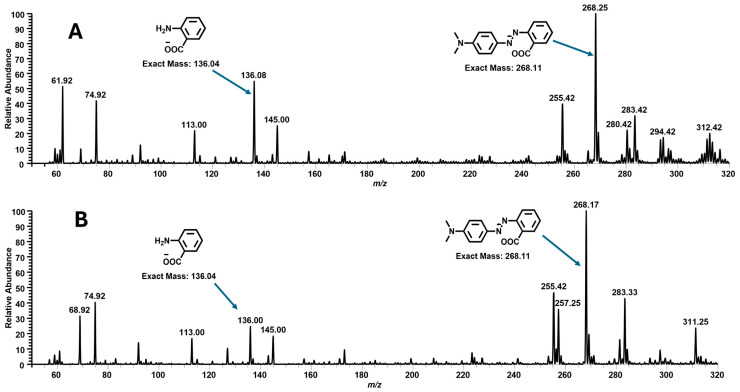
(**A**) MS spectrum of the photocatalytic reaction mixture of MR with 3000 equivalent hydrazine, 20 min after the starting point. (**B**) A part of MS spectrum of reaction mixture of MR with 6000 equivalents of hydrazine after 60 min reaction in the dark.

For compound MR, the reaction mixtures of different stages of both dark and photocatalytic reactions were also analyzed with a mass spectroscopic method, as shown in Figure 7.

The MR anion (268.11 calculated) peaks, which were shown in A (photocatalytic reaction) at 268.25 and in B (dark reaction) at 268.17, represent the dominant anion in the starting reaction mixtures of both reactions. As the intensity of this peak decreased, a new peak that was assigned to o-aminobenzoate (136.04 calculated) appeared in both A at 136.08 (photocatalytic reaction) and B at 136.00 (dark reaction). The intensity of this peak increased as the reaction proceeded, and was maximized when the reactions were completed. Unfortunately, the other amino product, N’N-dimethylaniline, was not sensitive enough to be determined with this method.

**Figure 8:**
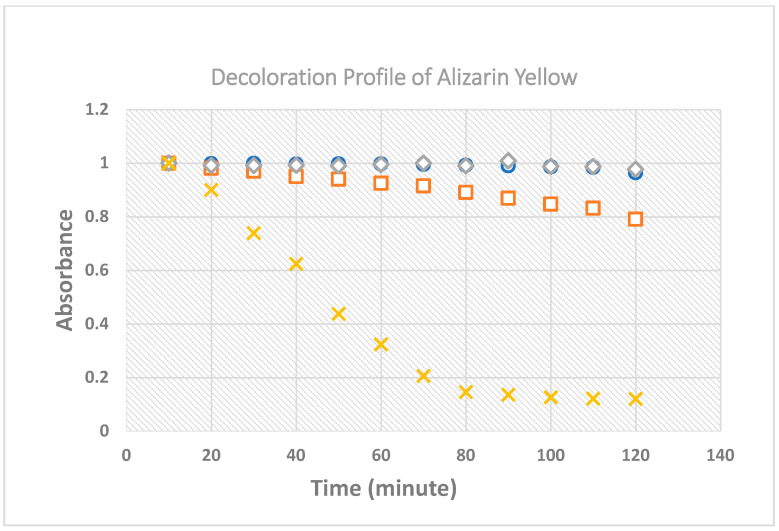
Decoloration rate of AY in hydrazine aqueous solution under different reaction conditions: ○ with no light and no catalyst; **□** with light but no catalyst; ◊ with catalyst but no light; and x with light and catalyst.

Alizarin yellow is a structural analog of MO and MR. However, it has a strong electron-withdrawing nitro group in contrast to a strong electron-donating N,N-dimethyl amino group in MO and MR. Therefore, AY was expected to behave significantly differently from MO and MR. The decoloration kinetic profiles of AY were determined in the same way as for MO and MR. The experimental results are presented in Figure 8.

**Scheme 2:**
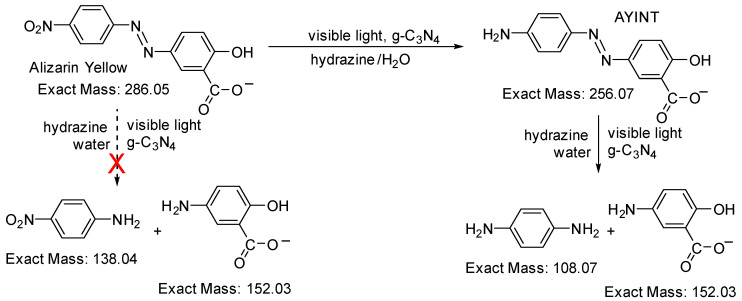
Photocatalytic and dark (when light not applied) reduction of AY by hydrazine and products.

The dark reaction of AY was extremely slow, as indicated by the slope of the apparent absorbance–time linear relationship. The reaction rate was calculated to be around 2.0 nmol/min based on the assay conditions. This rate is about 100 times slower than those of MO and MR. The light bleaching effect was also observed and its rate was comparable to those of MO and MR. The photocatalytic reaction rate of AY was calculated to be 1.3 μM/min, which is comparable to 1.3 μM/min for MO and 1.0 μM/min for MR. However, the photocatalytic reaction of AY is about 700 times faster than the uncatalyzed dark reaction, while the photocatalytic reactions of MO and MR are only about 5 times faster than the dark reaction. The mass spectroscopic analysis of the reaction mixtures revealed that AY decoloration followed a different pathway from those of MO and MR, which is shown in Scheme 2.

**Figure 9:**
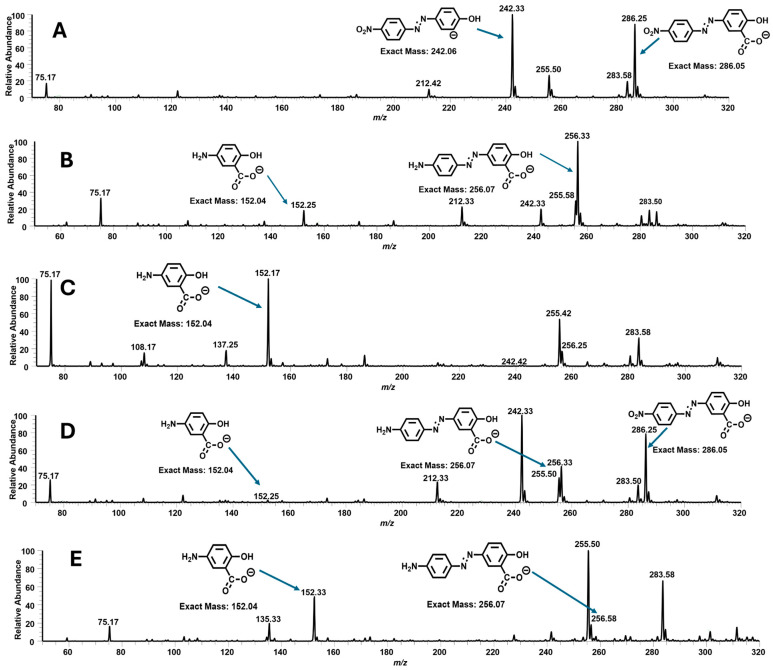
(**A**) MS spectrum of the starting reaction mixture of AY with hydrazine photocatalyzed by g-C_3_N_4_. (**B**) MS spectrum of the photocatalytic reaction mixture of AY with 1000 equivalent hydrazine, 20 min after the initial point. (**C**) MS spectrum of the photocatalytic reaction mixture of AY with 1000 equivalent hydrazine, 40 min after the initial point with a colorless solution formed. (**D**) MS spectrum of the reaction mixture of AY with 5000 equivalent hydrazine for 3 h in the dark. (**E**) MS spectrum of the reaction mixture of AY with 5000 equivalent hydrazine for 27 h in the dark.

As is illustrated in Scheme 2, the azo bond in AY was not reduced in the early stage like MO and MR to form two amines. Instead, it is the nitro group that was first reduced into an amino group to form a new azo bond containing an intermediate, here called AYINT (AY intermediate). This intermediate, AYINT, was then reduced to p-aminoaniline and 5-aminosalycylate. This reaction pathway was supported by the mass spectroscopic analysis of the reaction mixtures at different stages, as shown in Figure 9.

Mass spectrum A in Figure 7 indicates that the AY anion at 286.25 (286.05 calculated) is the dominant anion in the starting AY photocatalytic reaction mixture. The strong peak at 242.33 is assigned to the decarboxylated fragment (242.06, calculated) of AY generated in the analytic process. As the reaction proceeded, the intensity of the AY anion peak decreased and a new peak at 256.33 in mass spectrum B, assigned to AYINT (256.07 calculated), appeared and grew in intensity. The intensity of the AYINT peak maximized when the AY anion peak disappeared; that means all nitro groups are reduced to amino groups. At the same time, the peak of 5-aminosalycylate (152.04, calculated) as one of the azo-bond reduction products, appeared at 152.25 and started to grow. When the AYINT was consumed completely, that is, the reaction was completed, as indicated by the formation of a colorless suspension, 5-aminosalycylate (152.04, calculated) at 152.25 in mass spectrum C became the dominant anion in the reaction mixture. The dark reaction of AY was confirmed to follow the same pathway. Mass spectrum D of the dark reaction mixture clearly showed the stage when AY and the AYINT coexisted and the 5-aminosalycylate (152.04, calculated) peak at 152.25 appeared with a small intensity. 5-aminosalycylate was also the dominant product of the dark reaction, as shown in MS spectrum E. Furthermore, p-aminoaniline was not detected with this mass spectroscopic method due to its low sensitivity. In summary, the kinetic study results here indicated that azo compounds can be reduced to amines by hydrazine; g-C_3_N_4_ as a photocatalyst can catalyze this reduction reaction under visible light. The dark and photocatalytic reaction yielded the same product. It is also noteworthy that nitro groups in addition to azo bonds were also reduced in this process. The advantage of this chemical process over other methods [33,34,35,36,37] for conversion of azo groups to amines are that (1) the g-C_3_N_4_ catalysts are easy to make and environmentally friendly; (2) the precursors to g-C_3_N_4_ are cheap; (3) there is no involvement of organic solvents; and (4) the reaction conditions are mild. However, optimization of this reaction is to be carried out for practical application.

**Figure 10:**
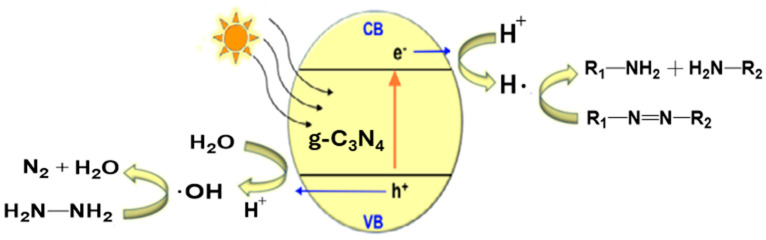
The proposed mechanism for the photocatalytic reduction of azo compound to amine by hydrazine under visible light.

Based on the observed results, we tentatively propose the mechanism in Figure 10 for this photocatalytic reaction. As g-C_3_N_4_ is a semiconductor, when irradiated by visible light, electrons in the valance band (VB) of g-C_3_N_4_ are excited to a conduction band (CB), and an electron and hole pair is generated with the positively charged hole in the valance band and an extra electron in the conducting band [38]. A proton from water combines with the electron to form a hydrogen radical associated to the catalyst. This hydrogen radical or hydrogen gas can then reduce the azo to two amines in multiple steps. The water can bind to the hole and generate a proton and a hydroxyl radical that associates to the catalyst. This hydroxyl radical or hydrogen peroxide will oxidize hydrazine to form water and nitrogen gas in multiple steps. Excess hydrazine in the reaction mixture is needed to protect the resulting amines from oxidation by the hydroxyl radical and the amines can be produced. The diimide of hydrazine is a possible intermediate in this process. Although it has not been observed, it cannot be excluded from the mechanism, because its following cleavage step might be too fast for it to be detected.

## 4. Conclusions

By simple pyrolysis of melamine at different temperatures, a series of g-C_3_N_4_ photocatalysts were prepared. The relative activity of the catalyst in the reduction of azo compounds by hydrazine was determined with the colorimetry method. It was found that the catalysts made at temperatures of 600 °C or above are most active. Our results demonstrated that azo compound reduction to amines by hydrazine can be catalyzed by g-C_3_N_4_ under visible light. The g-C_3_N_4_ photocatalyzed reaction of azo compounds with hydrazine led to the same product as the reaction carried out in the dark with no catalyst. Therefore, this photocatalytic reaction may provide a method to convert waste azo dye into amines that can act as precursors to azo dye again. In addition to the reduction of azo bonds to amines, a nitro group in the organic compound was also reduced to an amino group in this process, at an even faster rate than for the azo group. Therefore, this photocatalytic process may also provide a new method for the reduction of a nitro group into an amine.

## Figures and Tables

**Table 1:** The yield of the g-C_3_N_4_ catalysts and reaction conditions.

Temperature (°C)	Pyrolytic Time (Hour)	Melamine Used (mg)	g-C_3_N_4_ Produced (mg)
500	4	5005.9	2624.9
525	4	5009.3	2353.3
550	4	5003.1	2180.5
575	2	5005.9	1985.6
600	2	5005.4	1811.9
625	2	5033.5	1780.4
650	2	5030	1595.2
675	1	5038.2	1125.3
700	1	5032.5	897.2

## Data Availability

Data are available via request.
